# Effect of L-Ergothioneine on the metabolic plasma profile of the RUPP rat model of pre-eclampsia

**DOI:** 10.1371/journal.pone.0230977

**Published:** 2020-03-31

**Authors:** Aude-Claire Morillon, Rachel D. Williamson, Philip N. Baker, Douglas B. Kell, Louise C. Kenny, Jane A. English, Fergus P. McCarthy, Cathal McCarthy

**Affiliations:** 1 INFANT Research Centre, Cork, Ireland; 2 Department of Obstetrics and Gynecology, University College Cork, Cork, Ireland; 3 College of Life Sciences, University of Leicester, Leicester, United Kingdom; 4 Dept of Biochemistry, Institute of Integrative Biology, Faculty of Health and Life Sciences, University of Liverpool, Crown St, Liverpool, United Kingdom; 5 Novo Nordisk Foundation Centre for Biosustainability, Technical University of Denmark, Lyngby, Denmark; 6 Department of Women’s and Children’s Health, Institute of Translational Medicine, University of Liverpool, Liverpool, United Kingdom; 7 Department of Anatomy and Neuroscience, University College Cork, Cork, Ireland; 8 Department of Pharmacology and Therapeutics, University College Cork, Cork, Ireland; University of Mississippi Medical Center, UNITED STATES

## Abstract

**Introduction:**

Pre-eclampsia is a major cause of maternal and fetal mortality and morbidity worldwide. Its pathophysiology remains unclear, but mitochondrial dysfunction and oxidative stress have been implicated. L-Ergothioneine is a naturally occurring, water-soluble betaine, that has demonstrated antioxidant properties. Using the reduced uterine perfusion pressure (RUPP) rat model of pre-eclampsia, this study aimed to define the plasma metabolic profile following treatment with L-Ergothioneine.

**Methods:**

The effect of L-Ergothioneine (ET) treatment was explored using *in vivo* treatment in rats: Sham control (SC, n = 5), RUPP control (RC, n = 5), Sham +ET (ST, n = 5), RUPP +ET (RT, n = 5). Differential expression of plasma metabolites were obtained using untargeted liquid chromatography coupled to mass spectrometry. Statistical analysis was performed on normalised data comparing RC to SC, RT to RC, and RT to ST. Metabolites significantly altered (FDR < 0.05) were identified through database search.

**Results:**

We report significantly lower levels of L-palmitoylcarnitine in RC compared to SC, a fatty acyl substrate involved in beta-oxidation in the mitochondria. We report that a metabolite that has been associated with oxidative stress (Glutamylcysteine) was detected at significantly higher levels in RT vs RC and RT vs ST. Five metabolites associated with inflammation were significantly lower in RT vs RC and three metabolites in RT vs ST, demonstrating the anti-inflammatory effects of ET in the RUPP rat model of pre-eclampsia.

**Conclusions:**

L-Ergothioneine may help preserve mitochondrial function by increasing antioxidant levels, and reducing inflammatory responses associated with pre-eclampsia. This study shows the potential of L-Ergothioneine as a treatment for pre-eclampsia.

## Introduction

Pre-eclampsia (PE) is a hypertensive disorder of pregnancy, estimated to affect 3–5% of pregnancies worldwide annually, and leads to over 70,000 maternal deaths and 500,000 fetal and neonatal morbidities every year [1, 2]. The International Society for the Study of Hypertension in Pregnancy (ISSHP) defines pre-eclampsia as *de novo* hypertension after 20 weeks of gestation with or without proteinuria, presence of signs of liver dysfunction, maternal acute kidney injury, hemolysis or thrombocytopenia, neurological features, with or without presence of fetal growth restriction [2]. Pre-eclampsia is associated with preterm birth, iatrogenic or spontaneous, and fetal growth restriction [1], outcomes which are associated with an increased risk of respiratory and neurological disorders in the fetus. The pathophysiology of pre-eclampsia is still not fully understood, but poor placentation leading to exaggerated oxidative stress and systemic inflammatory response have been implicated [3, 4], as have dormant microorganisms [5, 6]. Deficient placental implantation and failed trophoblastic invasion of the spiral arteries lead to impaired blood perfusion between the myometrium and placenta, in turn leading to placental oxidative stress and an excessive production of reactive oxygen species (ROS) [3, 4, 7, 8]. ROS are reactive free radicals and other moieties which damage DNA, RNA and proteins; excessive production of ROS can overwhelm natural antioxidant defences and lead to oxidative stress [9]. Evidence of oxidative stress has been established in the well characterised reduced uterine perfusion pressure (RUPP) rat model of pre-eclampsia [10, 11]. Mitochondria are the main cellular source of ROS, and mitochondrial oxidative stress has recently been implicated in the pathophysiology of PE [11–13]. In their study, Vaka *et al*. provided novel evidence that placental ischaemia disrupted mitochondrial function with increased generation of mitochondrial-specific ROS. Furthermore, they showed that the levels of mitochondrial ROS were elevated in endothelial cells incubated with serum from RUPP rats compared to control rats. In addition, this study showed that endothelial cells incubated with serum from RUPP rats treated with mitochondrial specific antioxidant lead to a decrease in the amout of mitochondrial ROS produced, compared to untreated rats [11]. These recent studies indicate that directly targeting mitochondrial dysfunction could be a potential therapeutic treatment for pre-eclampsia.

L-Ergothioneine (ET) is a naturally occurring, water-soluble amino acid and a derivative of histidine, first discovered in the ergot fungus [14, 15]. Interestingly, the synthesis of ET has not been observed in animals or higher plants despite evidence of its accumulation in high concentration in animal and human tissues from dietary sources [16]; instead it exploits a solute carrier that has been seleced for this purpose [17]. Antioxidant properties of ET have been demonstrated by the reduction in markers of oxidative stress in blood samples taken from healthy human subjects, who were administered ET (5 or 25mg) once a day for 7 days [18]. The radical scavenging properties of ET are established, and this has led scientists to test it as a therapeutic agent in a range of pathologies, such as non-alcoholic fatty liver disease [19]. In addition, there is evidence that ET can accumulate in the mitochondrial fraction of hepatic cells in rats [20, 21].

A recent study investigated ET as a therapeutic agent to alleviate clinical symptoms of pre-eclampsia using the well established reduced uterine perfusion pressure (RUPP) rat model of pre-eclampsia, and determined that its therapeutic properties may in part results from a reduction in mitochondrial-specific ROS [22]. Here, we aimed to explore the plasma metabolic profile of L-Ergothioneine treatment in the RUPP rat model of pre-eclampsia.

## Materials and methods

### Materials and reagents

L-Ergothioneine was provided by Tetrahedron (Paris, France; www.tetrahedron.fr). Liquid chromatography grade methanol and formic acid were purchased from Fisher Scientific (Loughborough, UK). LC-MS glass vials and ultra-performance liquid chromatography (UPLC) column were purchased from Waters (Waters, Wexford, Ireland).

### Animals

University College Cork Biological Services Unit supplied and maintained the Sprague Dawley-timed pregnant rats used for the experiment. The animals had free access to food and tap water, and were maintained in a 12-hour light/dark cycle, at 21°C. All the procedures were performed in accordance with National Guidelines and the European Directive 2010/63/EU, under an authorization issued by the Health Products Regulatory Authority Ireland and approved by the Animal Ethics Committee of University College Cork (AE19130/P037).

### RUPP procedure

The RUPP procedure has been previously described [23, 24], and is a well-established surgical method used to model the link between hypertension in the pregnant rat and placental ischemia. Briefly, on gestational day (GD) 14, silver clips (0.2mm ID) were placed on the abdominal aorta (one clip) above the iliac bifurcation under isoflurane anaesthesia; this procedure induced the reduction of blood flow to the uteroplacental unit. An additional two clips (0.1mm ID) were carefully positioned around the right and left ovarian arteries. As a control, Sham surgery was performed by creating an incision, without placing any clips on either ovarian arteries or abdominal aorta. A chronic indwelling catheter was inserted into the carotid artery on GD18, and mean arterial blood pressure (MABP) was recorded in conscious animals on GD19.

### L-Ergothioneine *in vivo* experimental protocol

The effect of the ET treatment was explored using four experimental groups (Table 1): Sham control (SC, n = 5), RUPP control (RC, n = 5), Sham + ET (ST, n = 5), RUPP + ET (RT, n = 5). From GD11-GD19, ET was added to the drinking water at 25mg/kg/day, as reported in previous animal studies [25, 26]. Blood was collected on GD19 into EDTA vacutainers, these were centrifuged at 2000*g* and 2400*g* for 10 minutes at 4°C, plasma was removed and stored at -80°C for further analysis.

**Table 1:** Description of animal procedures and L-Ergothioneine *in vivo* experiment.

	Sham Control (n = 5)	RUPP Control (n = 5)	Sham + ET (n = 5)	RUPP + ET (n = 5)
Gestational day 11–19	Drinking water	Drinking water	Drinking water + ET	Drinking water + ET
Gestational day 14	Sham surgery	RUPP surgery	Sham surgery	RUPP surgery
Gestational day 19	Caesarean Section, Blood and Tissue Collection Experiment Endpoint			

ET: L-Ergothioneine, administered in drinking water at 25 mg/kg/day.

### Plasma sample preparation

Plasma samples were prepared in a randomised order. Samples were taken from -80°C storage, and left on ice until thawed. Metabolite extraction was performed based on the protocol by Dunn *et al* [27], and as follows: cold methanol (300μl) was added to plasma (100μl), vortex mixed, and centrifuged at 15,800*g* for 15 minutes at room temperature. Supernatant (200μl) was transferred in two separate properly labelled Eppendorf tubes, and placed in a centrifugal vacuum evaporator for 4 to 5 hours, without applying any heating. Samples were then reconstituted in water (100μl), vortex mixed and centrifuged at 15,800*g* for 15 minutes at room temperature. Pooled quality control (QC) samples were obtained by taking 20μl from each samples. Finally, supernatant of all samples and QC were transferred into LC-MS glass vials.

### UPLC-MS analysis

Samples were maintained at 4°C during the analysis, analysed in randomised order and in triplicate, on an ultra-high performance liquid chromatography (UPLC) Acquity system coupled to a Synapt G2-S quadrupole time-of-flight (Q-TOF) mass spectrometry system (Waters Corp, Wilmslow, UK). Data were acquired in both positive and negative electrospray ionisation modes (ESI+, ESI-). QC samples were analysed after every 10 samples injections. The chromatographic separation of the analytes was performed on a BEH C18 column (2.1x100mm, 1.7μm) maintained at 50°C. For both ESI+ and ESI-, the elution gradient used solvent A, a mix of water and formic acid at 0.1% (v/v), and solvent B, a mix of methanol and formic acid at 0.1% (v/v). In ESI+, injection volume was 4μl, and a 22 minutes gradient was applied at 0.40ml/min, as follows: initial condition at 0% of B for 1 minute, increased to 100% of B over 15 minutes, then maintained for 4 minutes, followed by a return to initial conditions of 0% of B in 2 minutes. In ESI-, injection volume was 7μl, and a 24 minutes gradient was applied as follows: initial condition at 0% of B for 2 minutes, increased to 100% of B over 15 minutes, then maintained for 5 minutes, followed by a return to initial conditions of 0% of B in 2 minutes.

Data were acquired in “resolution” MS^E^ mode [28, 29], from 50 to 1,500Da. Ion data for precursor (low energy) and fragment (high energy) were collected during the same acquisition, with a total cycle time of 0.2 seconds (0.1 second for each). Linear collision energy ramp (20-40eV) was applied for high energy. Capillary voltage was set to 3.0kV, sampling cone at 40V, extraction cone to 5V. The source temperature was set at 120°C, and desolvation temperature at 650°C. Desolvation gas flow rate was set at 800L/h, and cone gas at 50L/h. Mass calibration was performed using a sodium formate mix (Waters, Wexford, Ireland), recommended by the manufacturer before each batch analysis. Real time lock mass correction was performed using leucine enkephalin (LeuEnk, 1ng/μl) mix, injected at 10μL/min through a lock-spray probe and acquired every 30 seconds.

### Data processing

Data processing was performed using Progenesis QI version 2.4 (Nonlinear dynamics, Newcastle, UK), an appropriate pooled QC was selected as the reference to chromatographically align the data, and normalise to all compounds. Data were then peak picked, and considered the adducts corresponding with M+H, M+H-2H_2_O, M+H- H_2_O, M+NH_4_, M+Na, M+K, 2M+H (ESI+), or M-H, M-H_2_O-H, M-Na-2H, M+K-2H, M+Cl, M+FA-H, 2M-H (ESI-). Downstream statistical analysis was performed using the compound measurements exported from Progenesis QI.

### Statistical analysis

Statistical analysis of the data was performed on data normalised to all compounds (using Progenesis QI), and on the averaged intensities of the replicate measures. Data from each group were compared using Student’s T test (unpaired, two-tailed, unequal variance) in order to determine which metabolites were significantly altered between the groups. In addition, false discovery rate (FDR) correction using Benjamini- Hochberg [30], and mean fold change were calculated. Analysis was performed with Excel 2016 Software, and significance was accepted for FDR <0.05. Volcano plots and parallel coordinate plot were created using R statistical software [31] and the packages ggplot2 [32] and GGally [33].

### Putative annotation of metabolites

The exact mass of significant metabolites (FDR q-value <0.05) were putatively annotated [34] by database search using the Progenesis QI identification tool against the Human Metabolome Database (HMDB, version 4) [35]. The search parameters were set for an exact mass tolerance 5ppm for the precursor ion, and 10ppm for the fragment ion. Metabolites detected using UPLC-MS are frequently present multiple times due to fragmentation, multiple charging, chemical adduction, or dimerization. Identifications were reported for unique metabolites, after removing duplicates and metabolites of drugs or food, and after checking the retention time. The metabolites reported were grouped into chemical classes using HMDB, and the biological pathways or functions that they are involved with are listed, using HMDB, the small molecule pathway database (SMPDB) [36], and Kyoto encyclopedia of genes and genomes (KEGG) [37] (Table 2).

**Table 2:** Metabolites significantly altered (FDR q-value <0.05) and putatively annotated, for RUPP +ET compared to Sham +ET, RUPP +ET compared to RUPP Control, and RUPP Control compared to Sham Control.

Annotated metabolites	RUPP +ET vs Sham +ET		RUPP +ET vs RUPP Control		RUPP Control vs Sham Control		Chemical class	Chemical sub-class	Biological function/pathway
	FDR	FC	FDR	FC	FDR	FC			
9'-Carboxy-alpha-chromanol	1.29E-02	5.95	3.41E-02	2.37			Benzopyrans	1-benzopyrans	vitamin E biosynthesis
Glutamylcysteine OR Cysteinyl-Glutamate	1.37E-02	2.18	8.46E-03	11.11			Carboxylic acids and derivatives	Amino acids, peptides, and analogues	Glutathione metabolism
Glycyl-Serine OR Serylglycine	1.53E-02	2.02	3.14E-02	3.57			Carboxylic acids and derivatives	Amino acids, peptides, and analogues	Protein catabolism
Asparaginyl-Lysine OR Lysyl-Asparagine	1.69E-02	-1.28	4.32E-02	-1.21			Carboxylic acids and derivatives	Amino acids, peptides, and analogues	Protein catabolism
L-Phenylalanine	2.25E-02	6.02	1.95E-02	8.19			Carboxylic acids and derivatives	Amino acids, peptides, and analogues	Phenylalanine and Tyrosine Metabolism
L-Glutamic acid	4.72E-02	1.91	4.30E-02	2.64			Carboxylic acids and derivatives	Amino acids, peptides, and analogues	Alanine, Histidine, Cysteine Metabolism, Malate-Aspartate Shuttle, Ammonia Recycling
Histidinyl-Methionine OR Methionyl-Histidine	4.81E-02	2.27	4.10E-02	4.71			Carboxylic acids and derivatives	Amino acids, peptides, and analogues	Protein catabolism
Asymmetric OR symmetric dimethylarginine	2.29E-02	2.01					Carboxylic acids and derivatives	Amino acids, peptides, and analogues	Protein catabolism
Nonenoylglycine	3.36E-04	5.99					Carboxylic acids and derivatives	Amino acids, peptides, and analogues	Protein catabolism
Hydroxyprolyl-Isoleucine OR Hydroxyprolyl-Leucine	3.12E-03	2.11					Carboxylic acids and derivatives	Amino acids, peptides, and analogues	Protein catabolism
Alanyltryptophan OR Tryptophyl-Alanine	1.33E-02	-3.66					Carboxylic acids and derivatives	Amino acids, peptides, and analogues	Protein catabolism
N,O-Bis- (trimethylsilyl)phenylalanine	4.88E-02	3.45					Carboxylic acids and derivatives	Amino acids, peptides, and analogues	Protein catabolism
Pyridinoline	4.68E-02	2.04					Carboxylic acids and derivatives	Amino acids, peptides, and analogues	Protein catabolism
Histidinyl-Isoleucine OR Histidinyl-Leucine OR Isoleucyl-Histidine OR Leucyl-Histidine	2.51E-02	-1.39					Carboxylic acids and derivatives	Amino acids, peptides, and analogues	Protein catabolism
N-Heptanoylglycine OR 3-Hydroxyisoheptanoic acid OR Ethyl 2-hydroxyisovalerate	4.54E-02	4.92					Carboxylic acids and derivatives	Amino acids, peptides, and analogues	Protein catabolism
Histidinylhydroxyproline	4.87E-02	-1.19					Carboxylic acids and derivatives	Amino acids, peptides, and analogues	Protein catabolism
20-COOH-leukotriene E4			2.49E-02	1.56			Carboxylic acids and derivatives	Amino acids, peptides, and analogues	Arachidonic acid metabolism
Histidinyl-Tyrosine OR Asparaginyl-Tryptophan OR Tyrosyl-Histidine OR Tryptophyl-Asparagine			2.86E-02	-2.64			Carboxylic acids and derivatives	Amino acids, peptides, and analogues	Protein catabolism
Diphthamide			2.80E-02	-2.08			Carboxylic acids and derivatives	Amino acids, peptides, and analogues	
Asparaginyl-Methionine OR Methionyl-Asparagine			4.91E-02	12.39			Carboxylic acids and derivatives	Amino acids, peptides, and analogues	Protein catabolism
Cysteinyl-Glutamate OR Glutamylcysteine			3.73E-02	8.94			Carboxylic acids and derivatives	Amino acids, peptides, and analogues	Protein catabolism
Neuromedin N (1–4)					4.24E-03	-4.56	Carboxylic acids and derivatives	Amino acids, peptides, and analogues	Neuropepetide
Tyrosyl-Phenylalanine OR Phenylalanyl-Tyrosine					4.64E-02	-1.96	Carboxylic acids and derivatives	Amino acids, peptides, and analogues	Protein catabolism
L-Acetylcarnitine			8.22E-03	-1.53			Fatty Acyls	Fatty acid esters	Beta Oxidation of Very Long Chain Fatty Acids, Oxidation of Branched Chain Fatty Acids
L-Palmitoylcarnitine					2.06E-02	-10.35	Fatty Acyls	Fatty acid esters	Fatty acid Metabolism, Fatty acid Degradation
trans-Hexadec-2-enoyl carnitine OR Galactosylsphingosine OR Glucosylsphingosine	4.62E-02	3.32					Fatty Acyls	Fatty acid esters	Sphingolipid metabolism
Azelaic acid	1.69E-02	-2.29					Fatty Acyls	Fatty acids and conjugates	
5-Methyltetrahydrofolic acid (folic acid)	2.37E-02	1.27					Fatty Acyls	Fatty acids and conjugates	
(S)-3-Hydroxytetradecanoyl-CoA	2.73E-02	36.60					Fatty Acyls	Fatty acyl thioesters	Fatty Acid Elongation In Mitochondria, Fatty acid metabolism, Mitochondrial Beta-Oxidation of Long Chain Saturated Fatty Acids
3-Oxotetradecanoyl-CoA	3.89E-02	85.27					Fatty Acyls	Fatty acyl thioesters	Fatty Acid Elongation In Mitochondria, Fatty acid metabolism, Mitochondrial Beta-Oxidation of Long Chain Saturated Fatty Acids
S-(2-Methylpropionyl)-dihydrolipoamide-E	2.96E-02	3.66					Fatty Acyls	Fatty amides	Valine, leucine and isoleucine degradation
13-Hydroxyoctadecadienoic acid (13-HODE) OR other fatty acyls—11 hits	3.93E-02	2.86					Fatty Acyls	Lineolic acids and derivatives	Linoleic acid metabolism
20-Oxo-leukotriene E4	2.35E-02	2.88					Fatty Acyls	Eicosanoids	
Prostaglandin C2 OR 15-deoxy-PGD2 OR other prostaglandins—8 hits	3.84E-02	2.50					Fatty Acyls	Eicosanoids	Arachidonic Acid Metabolism
20-Trihydroxy-leukotriene-B4 OR Aldosterone OR Cortisone			2.98E-02	6.31			Fatty Acyls OR Steroids and steroid derivatives	Eicosanoids OR Hydroxysteroids	Arachidonic acid metabolism OR Steroid hormone biosynthesis
TG(48:3) OR other TG—31 hits	3.50E-02	1.48					Glycerolipids	Triradylcglycerols	
DG(36:3) - 2 hits			3.71E-02	1.27			Glycerolipids	Diradylglycerols	Glycerolipid metabolism
DG (35:0) - 3 hits			4.20E-02	1.89			Glycerolipids	Diradylglycerols	Glycerolipid metabolism
DG (39:1) - 3 hits					4.72E-02	-1.48	Glycerolipids	Diradylglycerols	Glycerolipid metabolism
LysoPA—2 hits	1.38E-02	-2.59					Glycerophospholipids	Glycerophosphates	Glycerolipid metabolism, Glycerophospholipid metabolism
PA—22 hits			2.89E-02	3.42			Glycerophospholipids	Glycerophosphates	Glycerolipid metabolism, Glycerophospholipid metabolism
PC—7 hits	3.75E-02	-11.38					Glycerophospholipids	Glycerophosphocholines	Glycerophospholipid metabolism, Arachidonic acid metabolism, Linoleic acid metabolism, alpha-Linolenic acid metabolism
PC—8 hits	1.49E-02	-1.39					Glycerophospholipids	Glycerophosphocholines	Glycerophospholipid metabolism, Arachidonic acid metabolism, Linoleic acid metabolism, alpha-Linolenic acid metabolism
PC—9 hits	4.26E-02	1.16					Glycerophospholipids	Glycerophosphocholines	Glycerophospholipid metabolism, Arachidonic acid metabolism, Linoleic acid metabolism, alpha-Linolenic acid metabolism
PC OR PE—34 hits	2.86E-02	-1.16					Glycerophospholipids	Glycerophosphocholines OR Glycerophosphoethanolamines	Glycerophospholipid metabolism, Arachidonic acid metabolism, Linoleic acid metabolism, alpha-Linolenic acid metabolism
PC OR PE—54 hits	2.21E-02	-2.00					Glycerophospholipids	Glycerophosphocholines OR Glycerophosphoethanolamines	Glycerophospholipid metabolism, Arachidonic acid metabolism, Linoleic acid metabolism, alpha-Linolenic acid metabolism
PE—4 hits	4.61E-02	1.22					Glycerophospholipids	Glycerophosphoethanolamines	Glycerophospholipid metabolism, Arachidonic acid metabolism, Linoleic acid metabolism, alpha-Linolenic acid metabolism
PC OR PE—18 hits	1.63E-02	15.43					Glycerophospholipids	Glycerophosphocholines OR Glycerophosphoethanolamines	Glycerophospholipid metabolism, Arachidonic acid metabolism, Linoleic acid metabolism, alpha-Linolenic acid metabolism
PC OR PE—37 hits	6.95E-03	-2.22					Glycerophospholipids	Glycerophosphocholines OR Glycerophosphoethanolamines	Glycerophospholipid metabolism, Arachidonic acid metabolism, Linoleic acid metabolism, alpha-Linolenic acid metabolism
PC OR PE—39 hits	2.96E-02	-1.40					GlycerophospholipidsV	Glycerophosphocholines OR Glycerophosphoethanolamines	Glycerophospholipid metabolism, Arachidonic acid metabolism, Linoleic acid metabolism, alpha-Linolenic acid metabolism
PE OR PC—39 hits			2.07E-02	3.93			Glycerophospholipids	Glycerophosphoethanolamines OR Glycerophosphocholines	Glycerophospholipid metabolism, Arachidonic acid metabolism, Linoleic acid metabolism, alpha-Linolenic acid metabolism
PE OR PC—58 hits			4.52E-02	3.53			Glycerophospholipids	Glycerophosphoethanolamines OR Glycerophosphocholines	Glycerophospholipid metabolism, Arachidonic acid metabolism, Linoleic acid metabolism, alpha-Linolenic acid metabolism
PE—3 hits			3.96E-02	2.75			Glycerophospholipids	Glycerophosphoethanolamines	Glycerophospholipid metabolism, Arachidonic acid metabolism, Linoleic acid metabolism, alpha-Linolenic acid metabolism
PE OR PC—16 hits			4.30E-02	-1.42			Glycerophospholipids	Glycerophosphocholines OR Glycerophosphocholines	Glycerophospholipid metabolism, Arachidonic acid metabolism, Linoleic acid metabolism, alpha-Linolenic acid metabolism
PE OR PC—36 hits			4.15E-02	-3.61	2.54E-02	9.29	Glycerophospholipids	Glycerophosphoethanolamines OR Glycerophosphocholines	Glycerophospholipid metabolism, Arachidonic acid metabolism, Linoleic acid metabolism, alpha-Linolenic acid metabolism
PA—20 hits			4.54E-02	3.83			Glycerophospholipids	Glycerophosphates	Glycerolipid metabolism, Glycerophospholipid metabolism
PA—24 hits			3.35E-02	1.43			Glycerophospholipids	Glycerophosphates	Glycerolipid metabolism, Glycerophospholipid metabolism
PS—10 hits			2.58E-03	1.63			Glycerophospholipids	Glycerophosphoserines	Glycine, serine and threonine metabolism, Glycerophospholipid metabolism
PS—16 hits			7.44E-03	1.63			Glycerophospholipids	Glycerophosphoserines	Glycine, serine and threonine metabolism, Glycerophospholipid metabolism
PS(36:8)			4.59E-02	1.99			Glycerophospholipids	Glycerophosphoserines	Glycine, serine and threonine metabolism, Glycerophospholipid metabolism
PC—4 hits			1.96E-02	-1.25			Glycerophospholipids	Glycerophosphocholines	Glycerophospholipid metabolism, Arachidonic acid metabolism, Linoleic acid metabolism, alpha-Linolenic acid metabolism
PC—20 hits			1.88E-02	-5.28			Glycerophospholipids	Glycerophosphocholines	Glycerophospholipid metabolism, Arachidonic acid metabolism, Linoleic acid metabolism, alpha-Linolenic acid metabolism
PE—7 hits			3.26E-02	-1.82			Glycerophospholipids	Glycerophosphoethanolamines	Glycerophospholipid metabolism, Arachidonic acid metabolism, Linoleic acid metabolism, alpha-Linolenic acid metabolism
PE—2 hits					2.30E-02	1.02	Glycerophospholipids	Glycerophosphoethanolamines	Glycerophospholipid metabolism, Arachidonic acid metabolism, Linoleic acid metabolism, alpha-Linolenic acid metabolism
Hydroxyphenylacetylglycine OR Hypoxanthine			4.12E-02	2.73			Imidazopyrimidines OR Carboxylic acids and derivatives	Purines and purine derivatives OR Amino acids, peptides, and analogues	Tyrosine metabolism OR Purine Metabolism
Decanoylcholine	2.00E-02	1.20					Organonitrogen compounds	Quaternary ammonium salts	
N-Acetyl-D-glucosaminyldiphosphodolichol	4.95E-02	4.80					Organooxygen compounds	Carbohydrates and carbohydrate conjugates	
3-Sialyl-N-acetyllactosamine OR 6-Sialyl-N-acetyllactosamine	3.03E-02	17.71					Organooxygen compounds	Carbohydrates and carbohydrate conjugates	
Quinone			1.19E-02	-1.28			Organooxygen compounds	Carbonyl compounds	Riboflavin Metabolism, Pyrimidine Metabolism
Spermidine monoaldehyde 1			4.52E-02	1.67			Organooxygen compounds	Carbonyl compounds	Arginine and proline metabolism, beta-Alanine metabolism, Glutathione metabolism, Bile secretion
N-Acetylneuraminic acid			3.43E-02	3.02			Organooxygen compounds	Carbohydrates and carbohydrate conjugates	Amino Sugar and Nucleotide Sugar Metabolism
Hydroxyphenyllactic acid	1.37E-02	2.18					Phenylpropanoic acids		
3-Hydroxy-4-methoxyphenyllactic acid OR other polyphenol metabolites—65 hits	9.39E-03	-1.73					Phenylpropanoic acids		
Formyl-CoA			4.64E-03	6.27			Purine nucleotides	Purine ribonucleotides	Oxidation of Branched Chain Fatty Acids, Glyoxylate and dicarboxylate metabolism
Ceramide (42:0)	2.27E-02	7.47					Sphingolipids	Ceramides	Sphingolipid metabolism
Ceramide (44:1)	1.49E-02	2.50					Sphingolipids	Ceramides	Sphingolipid metabolism
Ganglioside GA2 (30:0) OR Trihexosylceramide (30:1)	2.24E-02	37.00					Sphingolipids	Glycosphingolipids	Highly important in immunology
SM(32:1)	3.41E-02	1.20					Sphingolipids	Phosphosphingolipids	Sphingolipid metabolism, Sphingolipid signaling pathway, Necroptosis
Glucosylceramide (42:1)			4.08E-02	1.33	3.70E-02	-1.33	Sphingolipids	Glycosphingolipids	Sphingolipid metabolism
Ganglioside GM3 (32:0)			4.19E-02	-1.67			Sphingolipids	Glycosphingolipids	Highly important in immunology
Galabiosylceramide (44:1) OR Lactosylceramide (44:1)			3.12E-03	2.20			Sphingolipids	Glycosphingolipids	Sphingolipid metabolism
Glucosylceramide (36:1) OR Galactosylceramide (36:1)			1.98E-02	1.17			Sphingolipids	Glycosphingolipids	Sphingolipid metabolism
Ganglioside GM3 (40:2)			3.82E-02	3.23			Sphingolipids	Glycosphingolipids	Highly important in immunology
SM(44:1)					3.88E-02	1.16	Sphingolipids	Phosphosphingolipids	Sphingolipid metabolism, Sphingolipid signaling pathway, Necroptosis
3alpha,21-Dihydroxy-5beta-pregnane-11,20-dione	2.76E-02	2.41	3.71E-02	1.88	2.00E-02	-1.36	Steroids and steroid derivatives	Hydroxysteroids	Steroid hormone biosynthesis
17alpha,21-Dihydroxy-5beta-pregnane-3,11,20-trione OR Cortisol OR 18-Hydroxycorticosterone	3.06E-02	1.53					Steroids and steroid derivatives	Hydroxysteroids	Steroid hormone biosynthesis
Chenodeoxycholic acid sulfate OR Chenodeoxycholic acid 3-sulfate OR Ursodeoxycholic acid 3-sulfate	6.18E-03	2.29					Steroids and steroid derivatives	Bile acids, alcohols and derivatives	Fat digestion and absorption, Vitamin digestion and absorption
Glycoursodeoxycholic acid OR Deoxycholic acid glycine conjugate OR Chenodeoxycholic acid glycine conjugate OR Chenodeoxyglycocholic acid	3.48E-02	-7.61					Steroids and steroid derivatives	Bile acids, alcohols and derivatives	Fat digestion and absorption, Vitamin digestion and absorption
Estrone glucuronide	3.97E-02	2.09					Steroids and steroid derivatives	Steroidal glycosides	Steroid hormone biosynthesis
25-Hydroxyvitamin D3-26,23-lactone			4.77E-02	2.09			Steroids and steroid derivatives	Vitamin D and derivatives	Steroid hormone biosynthesis
Dehydroepiandrosterone 3-glucuronide OR Dehydroisoandrosterone 3-glucuronide			3.60E-02	3.54			Steroids and steroid derivatives	Steroidal glycosides	Steroid hormone biosynthesis
2-hydroxyethinylestradiol			2.51E-02	1.34			Steroids and steroid derivatives	Estrane steroids	Steroid hormone biosynthesis
5beta-Pregnanediol					4.32E-02	1.03	Steroids and steroid derivatives	Pregnane steroids	Steroid hormone biosynthesis
trans-Resveratrol 4'-O-glucuronide OR cis-Resveratrol 4'-O-glucuronide			1.17E-02	76.61			Stilbenes	Stilbene glycosides	Stilbenoid, diarylheptanoid and gingerol biosynthesis

FDR: false discovery rate q-value; FC: fold change.

## Results

### RUPP control vs sham control

**Fig 1:**
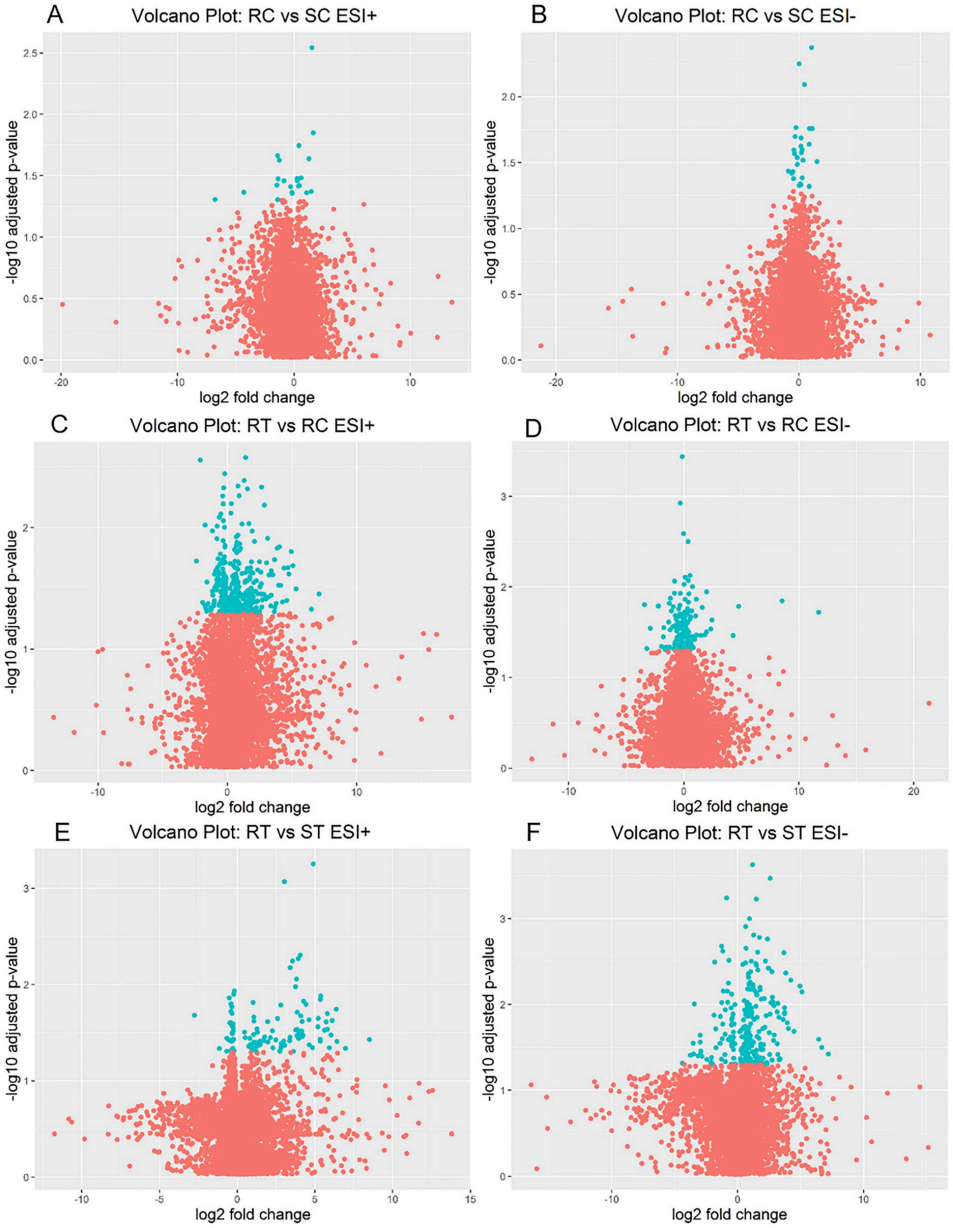
Volcano plot of metabolomics data, showing log2 fold change plotted against log10 of FDR q-values. (A) RUPP Control (RC) vs Sham Control (SC) in ESI+. (B) RUPP Control vs Sham Control in ESI-. (C) RUPP +ET (RT) vs RUPP Control in ESI+. (D) RUPP +ET vs RUPP Control in ESI-. (E) RUPP+ ET vs Sham+ET (ST) ESI+. (F) RUPP+ET vs Sham+ET ESI-. Features with FDR < 0.05 are plotted in blue.

Using UPLC-Q-TOF-MS, we compared metabolic profiles of RUPP rats (RC) and sham rats (SC) to identify metabolites that were altered in a rat model of pre-eclampsia. The statistical analysis comparing levels of metabolites detected in RC to SC showed that out of 15,024 detected features, 49 metabolites were significant (FDR q-value < 0.05); volcano plots are presented in Fig 1A and 1B. Among these 49 metabolites, 10 were annotated through database search, and belonged in 8 chemical classes (Table 2): two amino acids, peptides and analogues, one fatty acyl, one glycerolipid, two glycerophospholipids (GPL), two sphingolipids, and two steroids and steroid derivatives. All of these metabolites were detected at lower levels in RUPP control rats compared to sham rats, except for three (two GPL, and one steroid derivative). Interestingly, L-palmitoylcarnitine, a fatty acyl involved in mitochondrial respiration [38], was detected at lower levels in RUPP control group with a fold change of -10.35.

### RUPP+ET vs RUPP control

To identify metabolites that were altered following L-Ergothioneine treatment in the RUPP rat model of pre-eclampsia, we compared metabolic profiles of RUPP rats treated with ET (RT) to profiles of RUPP rats not treated (RC). Following statistical analysis, and out of 15,034 detected features, we reported 529 metabolites as significantly altered (FDR q-value < 0.05), volcano plots are presented in Fig 1C and 1D, and 45 metabolites were annotated through database search (Table 2). The metabolites annotated belonged to the following chemical classes: fourteen glycerophospholipids (GPL), eleven amino acids, peptides, and analogues, five sphingolipids, four steroid and steroid derivatives, two fatty acyls, two glycerolipids, one benzopyran, one imidazopyrimidine, three organooxygen compounds, one purine nucleotide, and one stilbene. The majority of these metabolites were detected at higher levels in the RUPP+ET group compared to RUPP control group. Out of the fourteen GPL detected, eight were phosphatidylcholines (PC) or phosphatidylethanolamines (PE), and are involved in arachidonic acid metabolism, linoleic acid metabolism, and alpha-linolenic acid metabolism. In addition, two metabolites involved in arachidonic acid metabolism were differentially detected between RT and RC: 20-Trihydroxy-leukotriene-B4, and 20-COOH-leukotriene E4. The second chemical class that showed the most altered metabolites was amino acids, peptides, and analogues, which are involved with protein catabolism, and two of these identified amino acids are involved with glutathione metabolism (Glutamylcysteine or Cysteinyl-Glutamate), and one with the malate-aspartate shuttle (L-Glutamic acid). In addition, two gangliosides were detected (Ganglioside GM3 (32:0) with a fold change of -1.67, and Ganglioside GM3 (40:2) with a fold change of 3.23).

### RUPP+ET vs Sham+ET

Similarly, we compared metabolic profiles of RUPP+ET to profiles of Sham+ET, and following statistical analysis of the 15,024 detected features, we reported 368 significant metabolites (FDR q-value < 0.05), volcano plots are presented in Fig 1E and 1F. 50 were annotated following database search (Table 2). The annotated metabolites belong in 10 chemical classes: fifteen amino acids, peptides, and analogues, ten glycerophospholipids (GPL), nine fatty acyls, five steroids and steroid derivatives, four sphingolipids, two organooxygen compounds, two phenylpropanoic acids, one benzopyran, one organonitrogen compound, and one glycerolipid. The majority of these metabolites (36) were detected at significantly higher levels in RUPP+ET group compared to Sham+ET group. Similar to the comparison of RUPP+ET to RC, among the 10 GPL detected as significanlty altered in RUPP+ET compared to Sham+ET, nine are PC or PE.

In addition, 20-Oxo-leukotriene E4, a metabolite of Leukotriene E4 (LTE4) following lipid oxidation, was detected at higher levels in RUPP+ET rats. Furthermore, two fatty acyls showed very high fold change in RUPP+ET compared to Sham +ET, (S)-3-Hydroxytetradecanoyl-CoA (FC 36.6) and 3-Oxotetradecanoyl-CoA (FC 85.27). Both of these fatty acids are involved with fatty acid metabolism, mitochondrial beta-oxidation of long chain saturated fatty acids, and fatty acid elongation in the mitochondria. In addition, Ganglioside GA2 (30:0) (or Trihexosylceramide (30:1)) was detected with a fold change of 37 in RUPP+ET compared to Sham+ET. Five of the amino acids and peptides detected as significantly altered between RUPP+ET and RC were also significantly altered in RUPP+ET compared to Sham+ET, most of which were detected at higher levels in RUPP+ET compared to Sham+ET, including L-Glutamic acid (FC 1.91) and Glutamylcysteine or Cysteinyl-Glutamate (FC 2.18).

**Fig 2:**
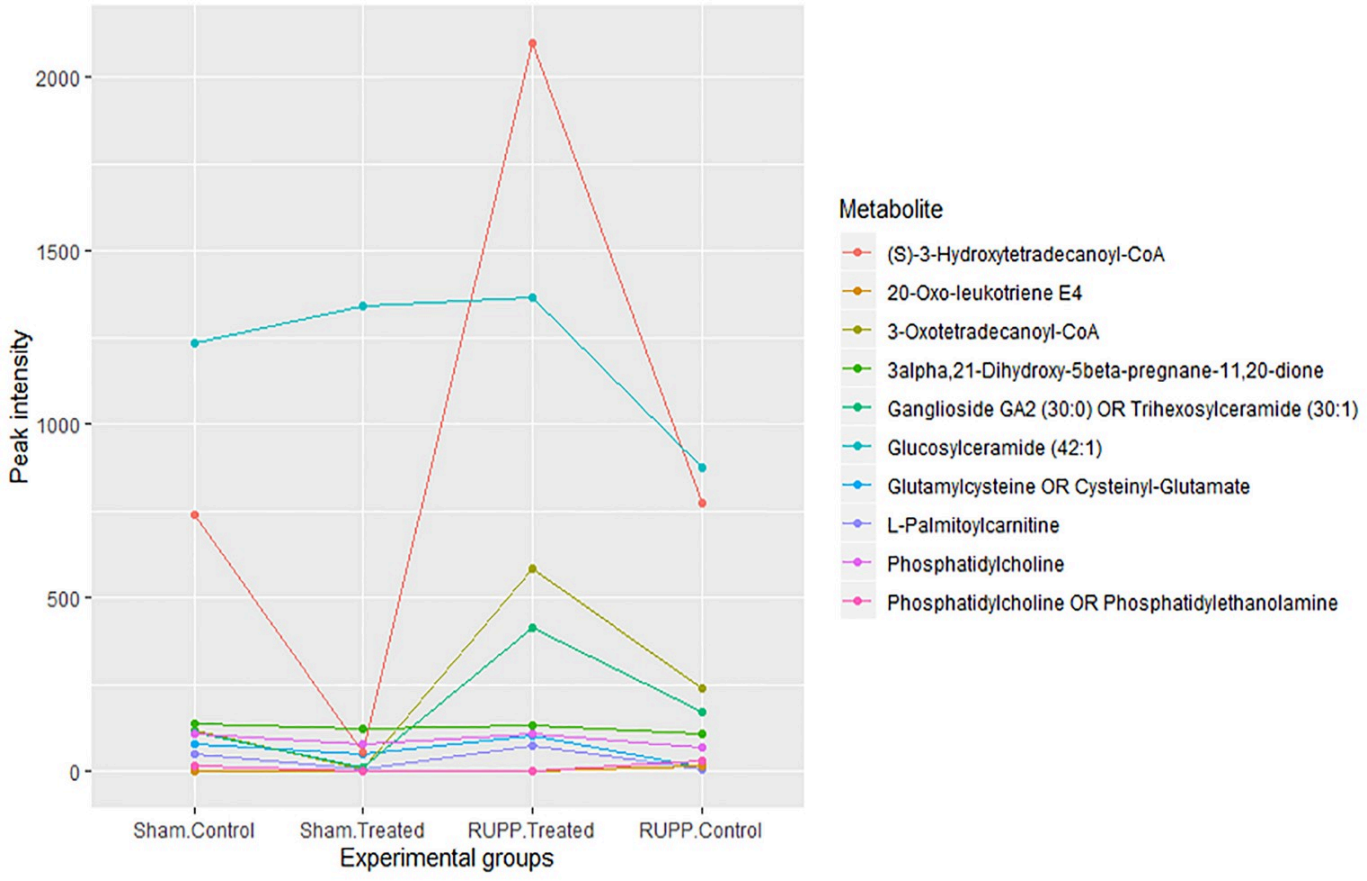
Parallel coordinate plot showing the averaged and normalised peak intensity of ten metabolites of interest in Sham Control, Sham +ET, RUPP Control and RUPP +ET rats.

Three metabolites were detected as significantly altered both in RUPP control vs Sham and in RUPP+ET vs RUPP control, with opposite fold changes: one GPL (a phosphatidylethanolamine or phosphatidylcholine), one sphingolipid (glucosylceramide (42:1)). One steroid derivative (3alpha,21-Dihydroxy-5beta-pregnane-11,20-dione) was detected as significantly altered in all comparisions: RUPP+ET compared to Sham+ET (FDR = 2.76E-02, FC 2.41), RUPP+ET compared to RUPP Control (FDR = 3.71E-02, FC 1.88), and RUPP Control compared to Sham Control (FDR = 2.00E-02, CF -1.36). A parallel coordinate plot showing the levels of 10 metabolites of interest in the fours experimental groups is shown in Fig 2, and demonstrates that for most of these metabolites, the levels in the RUPP+ET group are similar to those in the Sham Control group.

**Fig 3:**
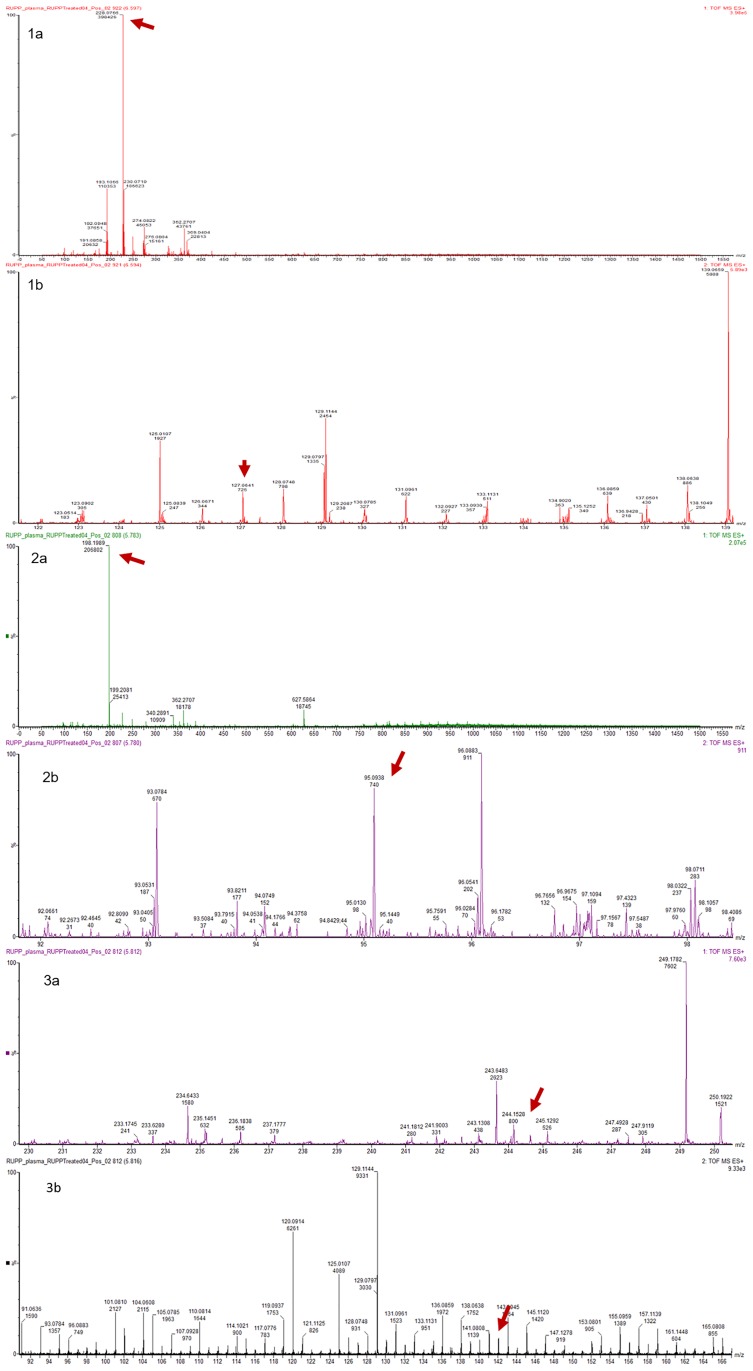
Mass spectra of L-Ergothioneine (1a, 1b), L-Hercynine (2a, 2b), and S-methyL-Ergothioneine (3a, 3b), detected in ESI+ mode using UPLC-Q-TOF-MS analysis. Spectra show m/z and intensity of peaks detected. 1a: MS1 scan showing parent ion of L-Ergothioneine at 228.0766 m/z, and 1b: MS2 scan showing its product ion at 127.0641 m/z; 2a: MS1 scan showing parent ion of L-Hercynine at 198.1989 m/z, and 2b: MS2 scan showing its product ion at 95.0938 m/z; 3a: MS1 scan showing parent ion of S-methyL-Ergothioneine at 244.1528 m/z, and 3b: MS2 scan showing its product ion at 141.0808m/z.

**Fig 4:**
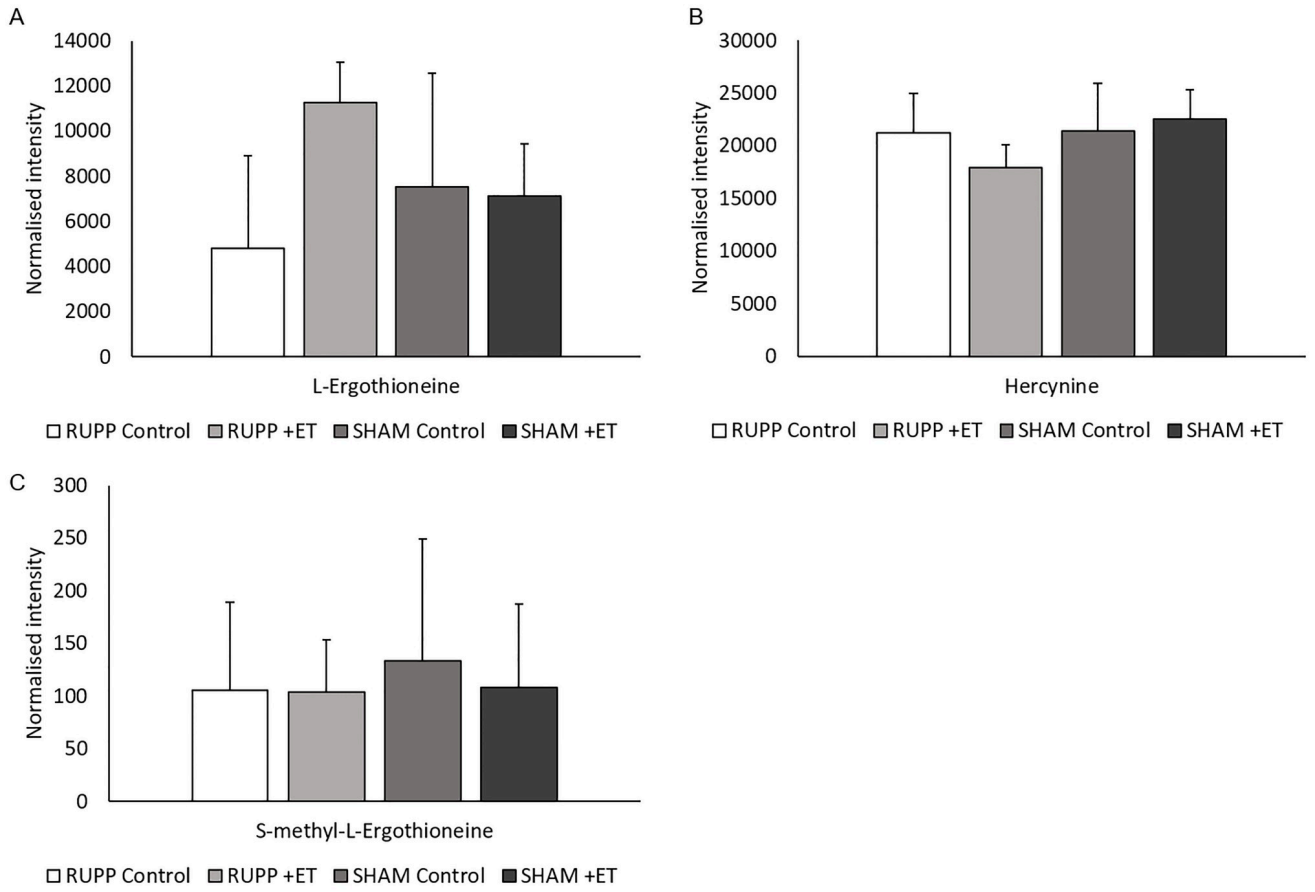
Plasma levels of L-Ergothioneine (A), Hercynine (B), and S-methyl- L-Ergothioneine (C). Levels are shown as normalised intensity as detected in ESI+ UPLC-Q-TOF-MS in plasma samples of RUPP Control, RUUP +ET, Sham Control and Sham +ET groups. No significant differences in levels observed between the groups. All values are expressed with standard error.

In addition, L-Ergothioneine and two of its metabolites (Hercynine, and S-methyl-L-Ergothioneine) were detected at similar relative levels in the plasma of all groups of animals in ESI+ (Figs 3 and 4). This presumably reflects the fact that it is excreted only very slowly so accumulates in the tissues.

## Discussion

The present study showed evidence of mitochondrial dysfunction in the plasma metabolite profile in the RUPP rat model of pre-eclampsia. This was evident from the lower levels of L-palmitoylcarnitine in RUPP+ET compared to RUPP Control (FC -10.35), which is associated with beta-oxidation in the mitochondria; from the higher levels of 3-Hydroxytetradecanoyl-CoA (FC 36.6) and 3-Oxotetradecanoyl-CoA (FC 85.27) in RUPP+ET compared to Sham+ET; and from the detection of several significantly altered sphingolipids, which are involved in various cellular processes, such as apoptosis and inflammation [39–41].

This study showed that L- palmitoylcarnitine was detected at significantly lower levels in RUPP Control compared to Sham Control rats (FC -10.35). Under physiological conditions, the mitochondria uses oxidative phosphorylation and the electron transport chain to produce ATP. However, it has been shown that pre-eclampsia is associated with mitochondrial dysfunction and oxidative stress [13, 42], and under such conditions, it is hypothesied that L- palmitoylcarnitine could be used as a mediator of mitochondrial respiration which could explain its lower levels in the RUPP Control rats, a model for pre-eclampsia, compared to healthy Sham Control rats. It has been shown that RUPP rats have increased evidence of mitochondrial dysfunction compared to sham controls [11, 22]. Williamson *et al*. have recently shown *in vivo* that mitochondrial-specific production of H_2_O_2_ in kidney tissue is increased in RUPP rats compared to sham control rats [43], which further demonstrated that mitochondrial dysfunction is associated with the pathophysiology of pre-eclampsia. This study also showed that the RUPP rats treated with ET (RUPP+ET) presented altered levels of fourteen glycerophospholipids (GPL) compared to RUPP rats (RC). Eight of these GPL are part of the arachidonic acid, linoleic acid, and alpha-linolenic acid metabolism pathways, which are used as precursors in the biosynthesis of several metabolites, such as eicosanoids or prostaglandins, involved in multiple cellular processes, including inflammation [39]. Here, we reported that five of these metabolites were detected at lower levels in RT rats compared to RC rats, demonstrating the anti-inflammatory effects of ET in the RUPP rat model of pre-eclampsia. In addition, L-glutamic acid was detected at higher levels in RT rats compared to RC. This amino acid is involved in the malate-aspartate shuttle system, or the malate shuttle, which is an essential system used by functional mitochondria allowing electrons to effectively move across the impermeable membrane between the cytoplasm and the mitochondrial matrix. Another metabolite of interest detected at higher levels in RUPP+ET rats is glutamylcysteine. This dipeptide is associated with glutathione metabolism, and a precursor of glutathione (GSH), an antioxidant and free radical scavenger [44]. Taken together, higher levels of glutamylcysteine in RUPP+ET rats indicates that ET can elevate antioxidant activity in the RUPP model of pre-eclampsia. One detected metabolite was annotated for glutathione, however it was not dected as significantly altered between the four experimental groups. We can hypothesise that L-Ergothioneine treatment in the RUPP rats helps alleviate oxidative stress by using a pathway alternative to glutathione, which is in line with the discovery of a solute carrier dedicated to the transport of L-Ergothioneine, solute carrier family 22 member 4 (SLC22A4) [17].

Furthermore, this study showed that two fatty acyls were detected as significantly altered and with very high fold change in RUPP+ET compared to Sham+ET, (S)-3-Hydroxytetradecanoyl-CoA (FC 36.6) and 3-Oxotetradecanoyl-CoA (FC 85.27). Both of these fatty acyls are involved with fatty acid metabolism, mitochondrial beta-oxidation of long chain saturated fatty acids, and fatty acid elongation in the mitochondria, and further the hypothesis of mitochondrial dysfunction during pre-eclampsia [11–13]. Two metabolites involved in arachidonic acid or linoleic acid metabolism pathways were significantly elevated in RUPP+ET compared to Sham+ET: Prostaglandin C2, 13-Hydroxyoctadecadienoic acid (13-HODE). In addition, Ganglioside GA2 (30:0) (or Trihexosylceramide (30:1)), was detected with a fold change of 37 in RUPP+ET compared to Sham+ET. Taken together, the significant alterations observed when comparing RUPP+ET to RUPP Control, and RUPP+ET to Sham Control tend to confirm the effects of L-Ergothioneine on oxidative stress and inflammatory response.

The low number of samples per rat groups (n = 5) is a limitation of the present study. However, the experiments were designed to obtain good quality data with the use of quality control samples and analysing the samples in a randomised order, as is recommended by metabolomics guidelines [45]. In addition, this study showed that metabolic profiling of plasma from RUPP rat model of pre-eclampsia was able to detect pathways altered by L-Ergothioneine treatment.

A previous metabolomics study aiming to identify early biomarkers of prediction of small for gestational age was performed on plasma samples taken from RUPP rats, human cord blood, and human maternal plasma sample collected at 15 weeks of gestation [46]. In this study, Horgan *et al*. aimed to validate in RUPP samples the panel of potential metabolite biomarker determined in human samples. Doing so, they confirmed eleven out of nineteen metabolites were also significantly altered in RUPP plasma samples. This panel includes an amino acid, a carnitine or vitamin D derivative, a fatty acid, a glycerolipid, and four glycerophospholipids. The majority of these metabolites were detected at higher levels in RUPP samples than in control rat samples. In our study, we observed that similar metabolite classes were significantly altered when comparing RUPP Control to Sham Control, and RUPP+ET to Sham+ET profiles. However, these metabolites show opposite trends in our study compared to what Horgan *et al*. reported. This said, these two studies were designed with major differences. Horgan *et al*. focused on small for gestational age and only reported metabolites in RUPP plasma samples, while in our study we focused on the pre-eclampsia phenotype and reported the metabolites significantly altered between RUPP control and Sham control, as well as after treatment with L-Ergothioneine by comparing profiles of RUPP+ET to RUPP control, and RUPP+ET to Sham+ET.

The strength of our data compared to previous studies investigating the pharmacological properties of L-Ergothioneine is that we examined its effects in the RUPP rat model of pre-eclampsia, and not only in healthy subjects as previously reported [18, 47]. The data presented in this study correlate with data previously reported by Cheah *et al*. [18] and Tang *et al*. [48], where both precursor ion and daughter fragment ions of L-Ergothioneine, Hercynine, and S-methyl-L-Ergothioneine were detected; however, there were no significant differences between the plasma levels in all groups in the present study. As previously reported, L-Ergothioneine accumulates in various organs and tissues in animals [16], including humans [18], especially in the liver, as well as in kidney, heart, spleen, intestines, eye, and brain tissues [48]. It has been reported that both brain tissue and urine from newborns contain L-Ergothioneine [49, 50], which implies this molecule is able to cross the blood-placental barrier [43]. A neuroprotective role for L-Ergothioneine has also been suggested by a number of *in vitro* and *in vivo* studies [43] however, more studies are needed to determine the effects on the newborns. Administration of pure L-Ergothioneine to animals [47] or healthy human subjects [18] has shown no adverse effects, and there is thus great potential to use this naturally occurring amino acid as a potential treatment for pre-eclampsia in a clinical trial.

In recent years, metabolomics techniques have become crucial tools in drug discovery [51]. A number of recent metabolomic alterations detected in pathologies, such as Alzheimer’s diseases [52], frailty [53], atherosclerosis [54], cardiovascular disease [21], or autism and schizophrenia [55], have led to new hypotheses on their aetiologies and new drug targets. The study of pathologies using metabolomics approaches allows for the detection of elevated or lower levels of specific metabolites, which are regulated by enzymes for either biosynthesis or used as a substrate in a biochemical reaction, and these enzymes can be targeted and new drug treatments can be tested [51].

In conclusion, L-Ergothioneine may mediate its therapeutics properties in part by preserving mitochondrial function, increasing antioxidant levels in addition to dampening the inflammatory response evident in pre-eclampsia. Furthermore, this study highlights the potential of L-Ergothioneine as a possible treatment for pre-eclampsia.

## Supporting information

S1 Table Compound measurement table for UPLC-MS positive mode analysis. (XLSX)

S2 Table Compound measurements table for UPLC-MS negative mode analysis. (XLSX)
